# A key to *Grouvellinus* Champion, 1923 from mainland China with descriptions of two new species (Coleoptera, Elmidae)

**DOI:** 10.3897/zookeys.623.9610

**Published:** 2016-10-11

**Authors:** Dongju Bian, Haibin Sun

**Affiliations:** 1 Institute of Applied Ecology, Chinese Academy of Sciences, Shenyang, 110016 China; 2 University of Chinese Academy of Sciences, Beijing 100049, China

**Keywords:** China, Elmidae, Grouvellinus, new species, taxonomy

## Abstract

A key to *Grouvellinus* Champion from mainland China is provided. Two new species of *Grouvellinus* are described from Jiangxi, China, *Grouvellinus orbiculatus* **sp. n.** and *Grouvellinus sagittatus* **sp. n.** Descriptions, diagnoses, and illustrations of the new species are given. Habitus photos of the nine known species are provided including the four species from Taiwan.

## Introduction

The generic name *Grouvellinus* was proposed by Champion, 1923 and is widely distributed in Oriental and Palearctic regions. Jäch (1984) made a revision of this genus based on material occurring in Himalaya and southeast Asia, re-describing seven species and reporting eight new species. Jäch and Kodada (1995) provided a check list of the Elmidae of China: four *Grouvellinus* species were mentioned as occurring in China, and five species were reported from bordering villages (Myanmar, Nepal). Subsequently, Jeng and Yang (1998) made a revision of *Grouvellinus* occurring in Taiwan and Japan, and three new species were reported from Taiwan. A total of 36 species of *Grouvellinus* are known so far (Brown 1981, Jäch et al. 2016, Jung et al. 2014).

According to Jäch et al. (2016), six species were known from mainland, China, and four species were reported from Taiwan. Jeng and Yang (1998) had already provided a key to *Grouvellinus* from Taiwan and Japan.

In this paper, a key for male to *Grouvellinus* from mainland China is provided and two new species of this genus are reported from Jiangxi, China. The genus *Grouvellinus* is reported from Jiangxi for the first time. Type material of the new species were deposited in Institute of Applied Ecology, Shenyang, Chinese Academy of Sciences
(IAECAS).Type materials of the known species were from Natural History Museum, Vienna (NHMW). Habitus photos of *Grouvellinus carus* Hinton, 1941 were provided by Dr. Maxwell V. L. Barclay (London).

## Material and methods

Specimens were examined with a Leica M205c stereomicroscope. Further details were studied under an Olympus BX51 compound microscope. Body length is the sum of pronotal and elytral lengths, body width means the broadest width of the elytra. The first strial interval means the sutural interval. Genitalia illustrations were drawn with the aid of a drawing tube. Male genitalia were placed in concentrated lactic acid in a cavity slide for at least several hours before they were examined. Photos were made with KEYENCE VHX-2000 – Super Resolution Digital Microscope System. Label data for holotypes, paratypes, and paralectotypes has been recorded verbatim, with lines on the same label separated by “/” and labels separated by “;”.

### Key to species reported from mainland China (male)

**Table:** 

1	Metasternum with two small pits on median suture	**2**
–	Metasternum without two small pits on median suture	**3**
2	Punctures on pronotum finer and sparser. Penis end up with an arrow, parameres gradually narrowed to apex (Figs 26–28)	***Grouvellinus sagittatus* sp. n.**
–	Punctures on pronotum bigger and denser. Penis end up with expanded rounded apex, and parameres distinctly narrowed at basal 1/3(Figs 23–25)	***Grouvellinus orbiculatus* sp. n.**
3	Body length no less than 2.5 mm	**4**
–	Body length less than 2.5 mm	**5**
4	Disc of metasternum with a pair of protuberances on each side of median suture at base (Fig. 18); penis sharply narrowed from base toward apex	***Grouvellinus hercules***
–	Disc of metasternum with a pair short carinae from basal 0.2 to 0.6 on each side of median suture (Fig. 20); penis sharply narrowed in distal 0.2	***Grouvellinus tibetanus***
5	Pronotum with a shallow oval impression medially on disc (Fig. 21)	***Grouvellinus carus***
–	Prontum without impression medially on disc	**6**
6	Pronotum with one median carina extending from base to basal 0.6; each elytral interval with a carina, carinae on intervals 3, 5, 7, 8 more developed (Fig. 11)	***Grouvellinus nepalensis***
–	Pronotum with a pair of short median carinae at base; elytra with strial intervals 7, 8 carinate (Fig. 15)	***Grouvellinus sinensis***

## Classification

### *Grouvellinus* Champion, 1923

*Grouvellinus* Champion, 1923: 168. Type species: *Macronychus caucasicus* Victor, 1839.

#### Grouvellinus orbiculatus sp. n.

Taxon classificationAnimaliaColeopteraElmidae

http://zoobank.org/ED10C0B1-C00D-4FD2-AC10-D397E7D578D9

Figures 1–2 , 23–25

##### Type material.

Holotype: male, “CHINA: Jiangxi / Ji’ an City, Suichuan/ County, Duiqian Town; 26°20'N 114°17'E, 228 / m, 2009.10.02, leg. / Bian & Tong (loc. 10)” (white label); “HOLOTYPE / Grouvellinus / orbiculatus sp. n.” (red label). Paratypes: 3 males, 4 females, the same data as holotype. 4 males, 2 females: “CHINA: Jiangxi / Ji’ an City, Suichuan / County, Caolin Town; 26°16'N 114°22'E, 228 / m, 2009.10.03, leg./ Bian & Tong (loc. 11)” (white label); “PARATYPE / Grouvellinus / orbiculatus sp. n.” (red label). 1 male: “CHINA: Jiangxi / Ganzhou City, Shangyou / County, Wuzhifeng Town; 25°57'N 114°05'E, 554 / m, 2009.10.05., leg. / Bian & Tong (loc. 12)”(white label); “PARATYPE / Grouvellinus / orbiculatus sp. n.” (red label).

##### Diagnosis.

This species is characterized by its small size (<2 mm), dense punctures on pronotum, sparse pubescence on the elytra, and the metasternum with two small pits on median suture; the penis end has a rounded expanded apex, and the parameres are distinctly narrowed at basal 1/3 in dorsal view.

##### Description.

**Figures 1–4.**
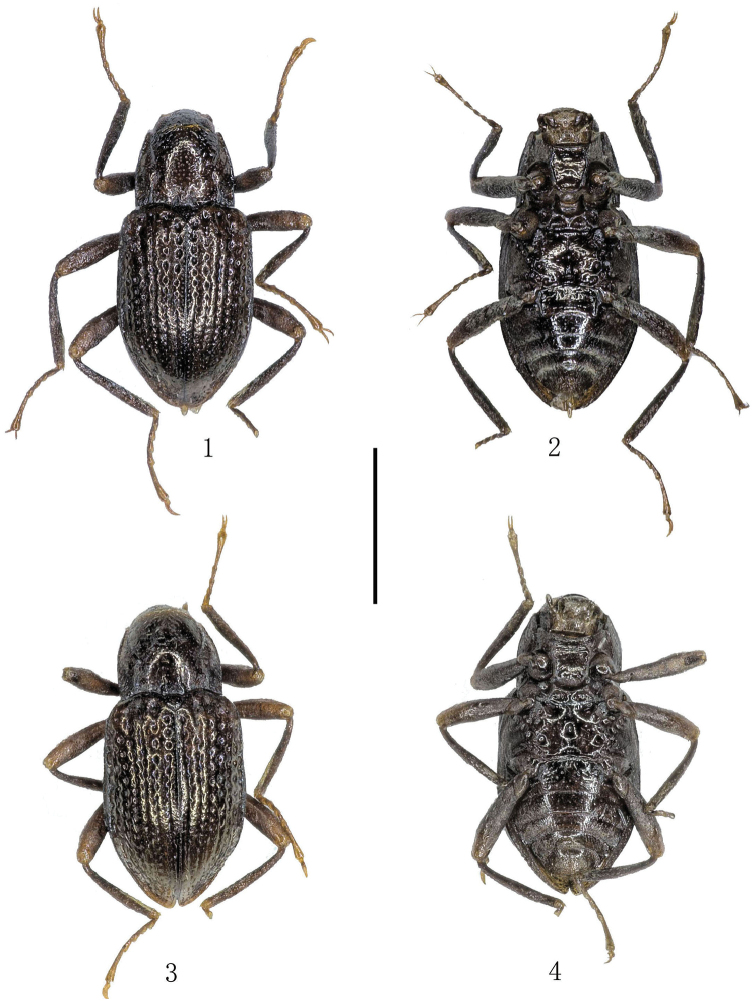
Habitus. **1–2** *Grouvellinus orbiculatus* sp. n., holotype **3–4** *Grouvellinus sagittatus* sp. n., holotype **1, 3** dorsal view **2, 4** ventral view. Scale bar: 1 mm. Photograph by Dongju Bian.

*Body* length 1.70 mm, body width 0.80 mm. Body shape elongate obovate, subparallel (Figs 1–2). Head black, pronotum and elytra brown, femora and tibiae dark brown. Antennae and tarsi yellowish brown.

*Head* smooth, covered with long pubescence; surface with small circular punctures sparsely and superficially impressed. Clypeus surface smooth, weakly punctate, lateral sides sparsely covered with long pubescence. Labrum smooth, weakly punctate and densely pubescent laterally.

*Pronotum* 1.2 times as broad as long, broadest at basal 2/5, surface smooth and shining, covered with adpressed pubescence; small circular punctures densely impressed. Sub-lateral carinae present on basal 2/5, feebly raised and feebly converging anteriorly; an oblique impression on each side extending from the end of carina to anterior angle. Base with two elongated oval impressions in front of anterior angles of scutellum.

*Elytra* 1.5 times as long as broad, 1.4 times as broad as pronotum; sides subparallel in basal 2/3, and then gradually narrowed to separated rounded apex. Strial punctures larger and deeper in basal half separated by 0.5–1.0 diameter; punctures becoming smaller and widely separated (2–5 times diameters); intervals slightly convex, and each interval with one or two rows of small punctures and pubescence; intervals 2–4 slightly elevated at base; interval 8 carinate.

Process of *prothoracic ventrite* approximately1/3 as broad as pronotum, distinctly rimmed; basal 2/3 disc smooth and shining, slightly elevated, apex somewhat truncate (Fig. 2). Disc of metasternum broadly impressed, surface smooth and shining, weakly punctate, and two small pits present on median suture; sub-lateral area of metasternum densely pubescent; metasternum with two rows of punctures on each side, one is oblique consisting of very large punctures which begin at anterior margin laterally and end up in front of metacoxae, the other one consisting of moderate-sized punctures is in front of posterior margin. Middle regions of ventrites I–IV smooth and shining, with small punctures sparsely distributed, and sub-lateral regions densely pubescent; middle region of ventrite V at basal 1/3 is similar to ventrites I–IV, elsewhere on ventrite V densely pubescent and sparsely granulate.

*Male genitalia* (Figs 23–25) long and slender, about 690 µm in length. Penis distinctly surpasses parameres, gradually narrowed from base to distal portion, apex expanded ending up with broadly rounded apex; ventral sac with lateral sides weakly sclerotized, surpassing the parameres. Parameres robust at base, distinctly narrowed at basal 1/3 in dorsal view.

*Female* similar to male, but disc of metasternum not broadly impressed, slightly elevated.

##### Distribution.

China: Jiangxi.

##### Etymology.

The specific name comes from the Latin word “orbiculatus” and refers to the rounded apical portion of penis.

##### Remarks.

*Grouvellinus orbiculatus* sp. n. is similar to *Grouvellinus sagittatus* sp. n. in body size, habitus, interval 8 on elytra with a carina, and two small pits on median suture of metasternum, but can be distinguished from the latter by its denser punctures on pronotum, penis ends with expanded rounded apex, and parameres distinctly narrowed at basal 1/3.

#### Grouvellinus sagittatus sp. n.

Taxon classificationAnimaliaColeopteraElmidae

http://zoobank.org/609F02D9-A6C8-4F29-B2DF-A8EEBB5903F9

Figures 3–4 , 26–28

##### Type materials.

Holotype, male: “China: Jiangxi / Ganzhou, Longnan / County, Jiulianshan; 24°37'N 114°32'E, 560 / m 2009.10.10, Leg. Bian / & Tong (16)”(white label); “HOLOTYPE / Grouvellinus / sagittatus sp. n.”(red label). Paratypes: 2 males, 2 females: “CHINA: Jiangxi, / Ganzhou City, Longnan / County, Jiulianshan; Downstream of loc. 16; / 2009.10.10; leg. Bian & / Tong (loc. 17)” (white label); “PARATYPE / Grouvellinus / sagittatus sp. n.” (red label).

##### Diagnosis.

This species is characterized by its small size (<2 mm), dorsum sparsely pubescent, and metasternum with two small pits on the median suture; penis ends with an arrow, and parameres gradually narrowed from base towards apex.

##### Description.

*Body* length 1.75 mm, body width 0.80 mm. Body shape elongate obovate, subparallel (Figs 3–4). Dorsal surface brown to dark brown, with strong bronze luster; elytra paler than pronotum. Ventral surface brown to dark brown, femora and tibiae brown, antennae, mouth parts, and tarsi yellowish-brown.

*Head* smooth and shining, covered with long pubescence; small circular punctures sparsely impressed. Clypeus surface smooth, sparsely punctate and pubescent; labrum smooth, surface of disc weakly punctate and pubescent, and densely pubescent laterally.

*Pronotum* 1.2 times as broad as long; broadest at basal 2/5. Surface smooth and shining, accompanied with long adpressed pubescence; small circular punctures sparsely and superficially impressed. Basal sub-lateral carinae present on basal 2/5, but only slightly raised, feebly converging anteriorly; an oblique impression on each side extending from apical end of carina to anterior angle; base with two elongate oval impressions in front of anterior angles of scutellum.

*Elytra* 1.6 times as long as broad, 1.4 times as broad as pronotum; sides subparallel in basal 2/3, and then tapering to separated rounded apex. Strial punctures larger and deeper on disc, but becoming finer and shallower on apical declivity; intervals convex, smooth and shining, each interval with one or two rows of small punctures and pubescence; intervals 2–4 slightly elevated at base; interval 8 carinate.

Process of *prothoracic ventrite* approximately1/3 as broad as pronotum, subquadrate, strongly rimmed, with transverse, smooth elevation at base; surface almost without punctures and pubescent on disc (Fig. 4). Disc of metasternum flat, smooth and shining, sparsely punctate, with two small pits on median suture, one is longitudinal which on the middle of metasternum, and the other one is transverse which is in front of the posterior margin; sub-lateral region densely pubescent, with two rows of coarse punctures on each side, one is oblique with very large punctures which begin at anterior margin laterally and end up in front of the metacoxae, the other one consisting of moderate-sized punctures is in front of posterior margin. Middle regions of ventrites I–IV smooth and shining, with few small punctures; sub-lateral regions densely pubescent; distal 2/3 of ventrite V granulate.

*Male genitalia* (Figs 26–28): long and slender, 605 µm in length; penis extends beyond parameres, distinctly narrowed from base toward apex, arrowed at the tip; ventral sac developed, longer than parameres. Parameres gradually narrowed from base to apex in dorsal view.

*Females* similar to males, but disc of metasternum not flat, distinctly elevated.

##### Distribution.

China: Jiangxi.

##### Etymology.

The specific name comes from the Latin word “sagittatus” meaning “arrow-shaped” and refers to the inflated apical portion of penis.

##### Remarks.

*Grouvellinus sagittatus* sp. n. is similar to *Grouvellinus orbiculatus* sp. n. in body size, habitus, interval 8 with a carina, and two small pits on median suture, but can be distinguished from the latter by sparser punctures on pronotum, penis ends with an arrow, and parameres gradually narrowed from base to apex in dorsal view.

#### Grouvellinus babai babai

Taxon classificationAnimaliaColeopteraElmidae

Nomura, 1963

Figures 5–6

Grouvellinus babai babai Nomura, 1963: 55. TL: China, Taiwan.

##### Distribution.

China: Taiwan.

##### Material examined.

1 male: “Taiwan, Taipei / Wanli 13. 8. 90 / M.L. Jeng (111)”.

**Figures 5–8.**
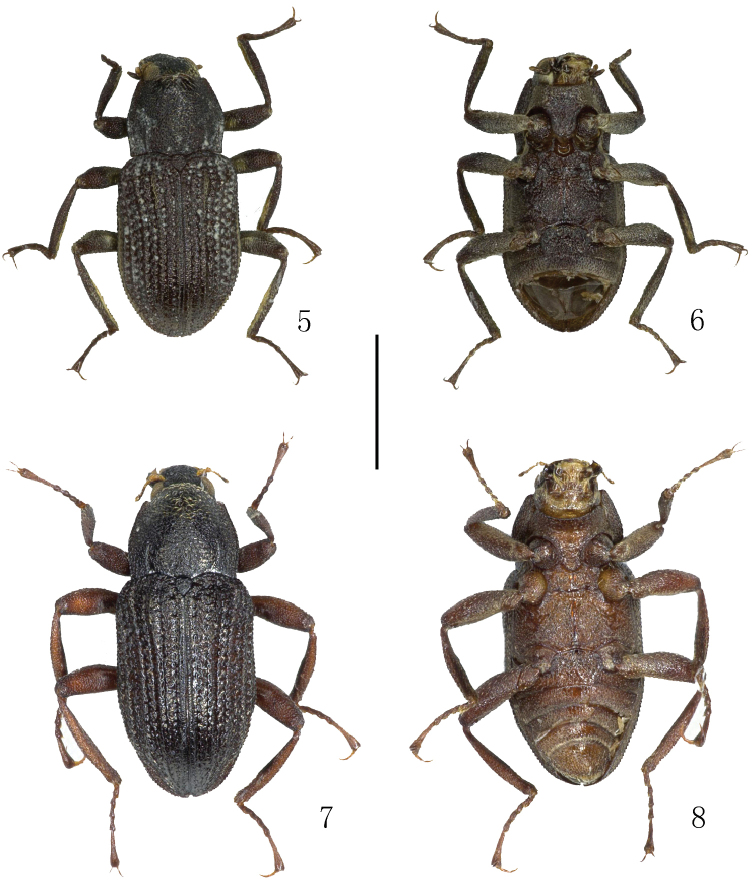
Habitus. **5–6** *Grouvellinus babai babai* Nomura, 1963 **7–8** *Grouvellinus hydropetricus* Jeng & Yang, 1998, paratype **5, 7** dorsal view **6, 8** ventral view. Scale bar: 1 mm. Photograph by Dongju Bian.

#### Grouvellinus chinensis

Taxon classificationAnimaliaColeopteraElmidae

Mařan, 1939

Grouvellinus chinensis Mařan, 1939: 42. TL: China, Sichuan.

##### Distribution.

China: Sichuan.

##### Remarks.

This species was described by Mařan in 1939 based on a female specimen. The type specimen was deposited in National Museum, Prague, Czech Republic (NMPC). Dr. Martin Fikáček was contacted, but he did not find the type specimen in NMPC, and also did not find any record of rent loans. According to the original description, this species was 3.0 mm long, 1.3 mm wide, strial intervals transversely wrinkled on elytra, and intervals 3, 5, 7, 8 carinate, while the two new species were less than 2.0 mm long, strial intervals smooth and shining on elytra, and interval 8 carinate. This species was also not included in the key because the key was only for males.

#### Grouvellinus hydropetricus

Taxon classificationAnimaliaColeopteraElmidae

Jeng & Yang, 1998

Figures 7–8

Grouvellinus hydropetricus Jeng & Yang, 1998: 533. TL: China, Taiwan.

##### Distribution.

China: Taiwan.

##### Material examined.

Paratype: 1 male: “Taiwan 8.IX. 93 / Taipei,Wulei / leg. M. L. Jeng” (white label); “PARATYPUS / Grouvellinus / hydropetricus n. sp. / des Jeng & Yang, 1993” (red label).

#### Grouvellinus montanus

Taxon classificationAnimaliaColeopteraElmidae

Jeng & Yang, 1998

Figures 9–10

Grouvellinus montanus Jeng & Yang, 1998: 530. TL: China, Taiwan.

##### Distribution.

China: Taiwan.

##### Material examined.

Paratype: 1male: “Ilan Taiwan / Chiduan / 29 III. 1990 / Jeng M. L. leg.” (white label); “PARATYPUS / Grouvellinus / montanus / JENG & YANG / des. Jeng & Yang” (red label).

**Figures 9–12.**
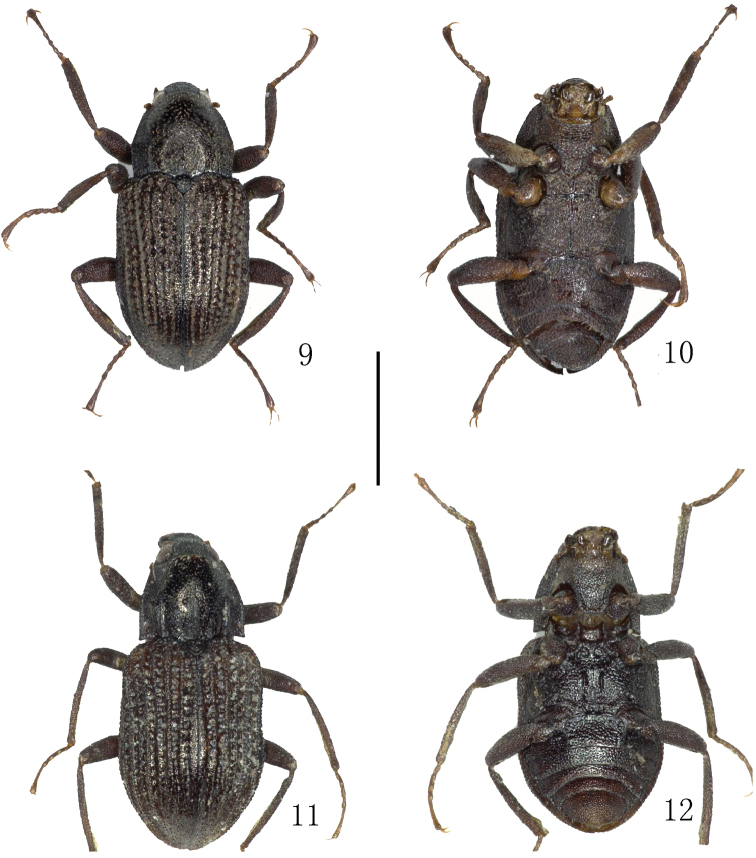
Habitus. **9–10** *Grouvellinus montanus* Jeng & Yang, 1998, paratype **11–12** *Grouvellinus nepalensis* Delève, 1970 **9, 11** dorsal view **10, 12** ventral view. Scale bar: 1 mm. Photograph by Dongju Bian.

#### Grouvellinus nepalensis

Taxon classificationAnimaliaColeopteraElmidae

Delève, 1970

Figures 11–12

Grouvellinus nepalensis Delève, 1970: 321. TL: China, Nepal.

##### Distribution.

China: Xizang; Nepal.

##### Material examined.

1 male: “Nepal, 28.2.31 / Tibetan. Grenze / leg. M. Jäch”.

#### Grouvellinus pilosus

Taxon classificationAnimaliaColeopteraElmidae

Jeng & Yang, 1998

Figures 13–14

Grouvellinus pilosus Jeng & Yang, 1998: 527. TL: China, Taiwan.

##### Distribution.

China: Taiwan.

##### Material examined.

Holotype: 1 male: “Taiwan 16. VIII. 90 / Taipei / Sanshah: Yomushi / leg. M. L. Jeng”; “HOLOTYUS / Grouvellinus / pilosus sp.n. / des. Jeng & Yang 1993” (red label).

**Figures 13–16.**
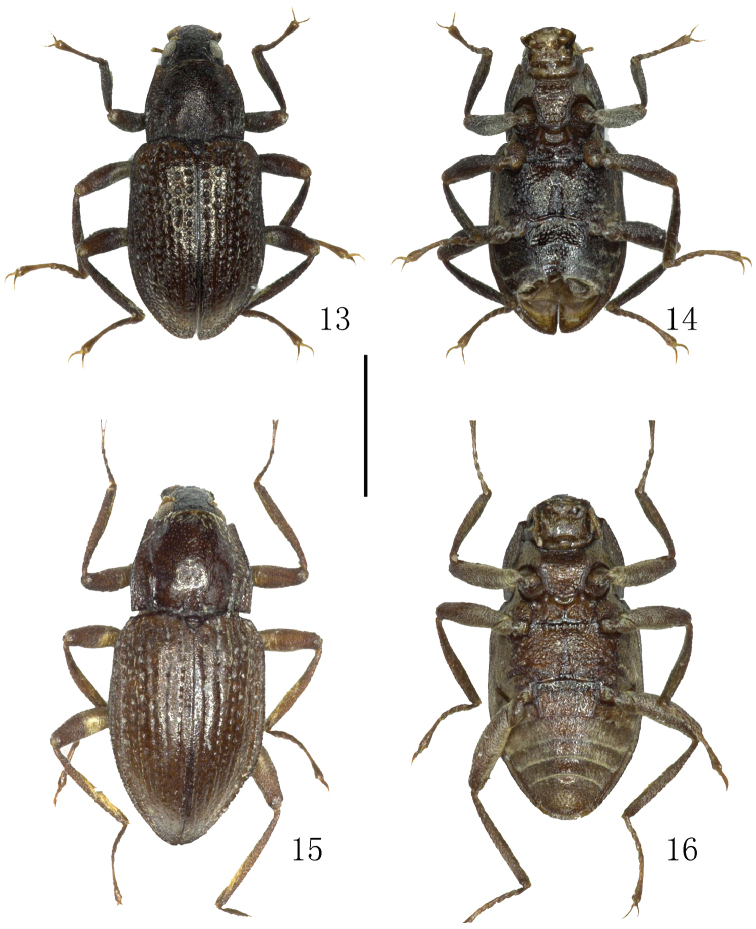
Habitus. **13–14** *Grouvellinus pilosus* Jeng & Yang, 1998, holotype **15–16** *Grouvellinus sinensis* Grouvelle, 1906, paralectotype **13, 15** dorsal view **14, 16** ventral view. Scale bar: 1 mm. Photograph by Dongju Bian.

#### Grouvellinus sinensis

Taxon classificationAnimaliaColeopteraElmidae

Grouvelle, 1906

Figures 15–16

Grouvellinus sinensis Grouvelle, 1906: 126. TL: China, Yunnan.

##### Distribution.

China: Yunnan.

##### Material examined.

Paralectotypes: 1male, 1 female: “Yun-nan, Chine / Grouvelle / 1906” (white label); “PARALECTOTYPUS / *Microdes* / *sinensis* Grouvelle / des. M. A. Jäch” (red label).

#### Grouvellinus hercules

Taxon classificationAnimaliaColeopteraElmidae

Jäch, 1984

Figures 17–18

Grouvellinus hercules Jäch, 1984: 113. TL: Nepal.

##### Distribution.

China: Xizang; Nepal.

##### Material examined.

1 male: “Nepal, 35 Km NW Pokhara / Ulleri 2000m / leg. Wewalka 5.5. 1984 (N4)”.

**Figures 17–20.**
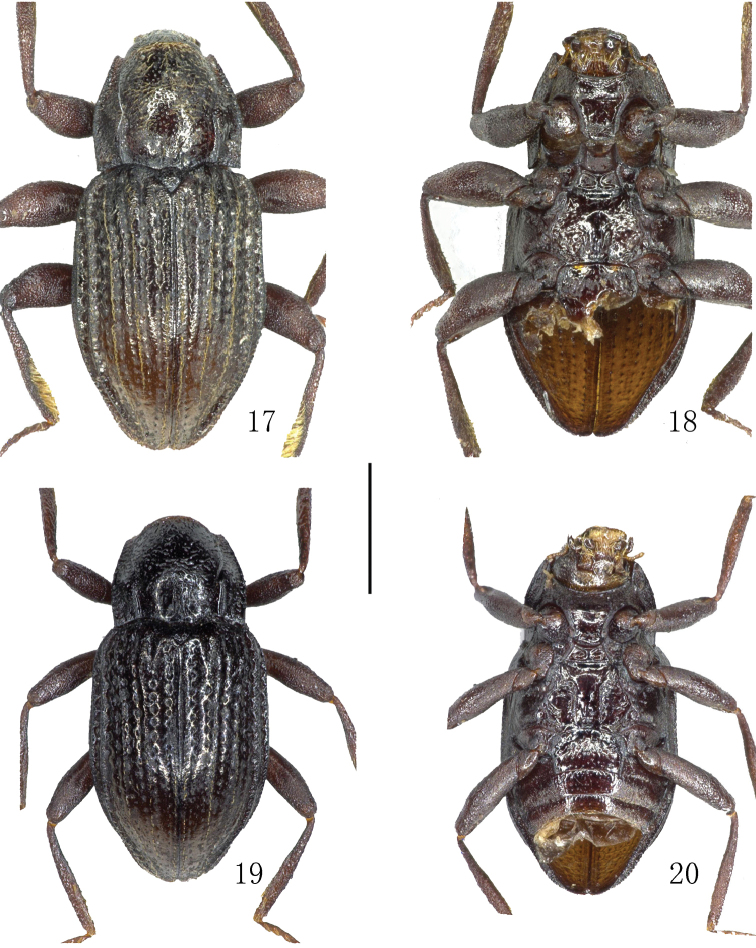
Habitus. **17–18** *Grouvellinus hercules* Jäch, 1984 **19–20** *Grouvellinus tibetanus* Jäch, 1984, paratype **17, 19** dorsal view **18, 20** ventral view. Scale bar: 1 mm. Photograph by Dongju Bian.

#### Grouvellinus tibetanus

Taxon classificationAnimaliaColeopteraElmidae

Jäch, 1984

Figures 19–20

Grouvellinus tibetanus Jäch, 1984 : 114. TL: Nepal.

##### Distribution.

China: Xizang; Nepal.

##### Material examined.

Paratype: 1 male: “Nepal, 1.3.81. / Tibetan. Gregze /leg. M. A. Jäch, N 31/ Tatopani” (white label); “Para / TYPUS” (red label).

#### Grouvellinus carus

Taxon classificationAnimaliaColeopteraElmidae

Hinton, 1941

Figures 21–22

Grouvellinus carus Hinton, 1941: 71. TL: China, Fujian.

##### Distribution.

China: Fujian.

##### Remarks.

This species was described by Hinton in 1941, and the type specimens were deposited in Natural History Museum, London. Dr. Maxwell V. L. Barclay (London) provided the habitus photos of the holotype which were taken by Keita Matsumoto (London). According to the photos and the original description, the following differences between the *Grouvellinus carus* and the two new species are noted: *Grouvellinus carus* has an oval impression medially on pronotum, surface of head with a few granules, metasternum without two small pits on median suture, and all of ventrites II to V densely pubescent and sparsely granulate, while the two new species are without impressions medially on pronotum, head smooth and shining, without granules, metasternum with two small pits on median suture, and middle regions of ventrites I to IV smooth and shining, without granules, not densely pubescent.

**Figures 21–22.**
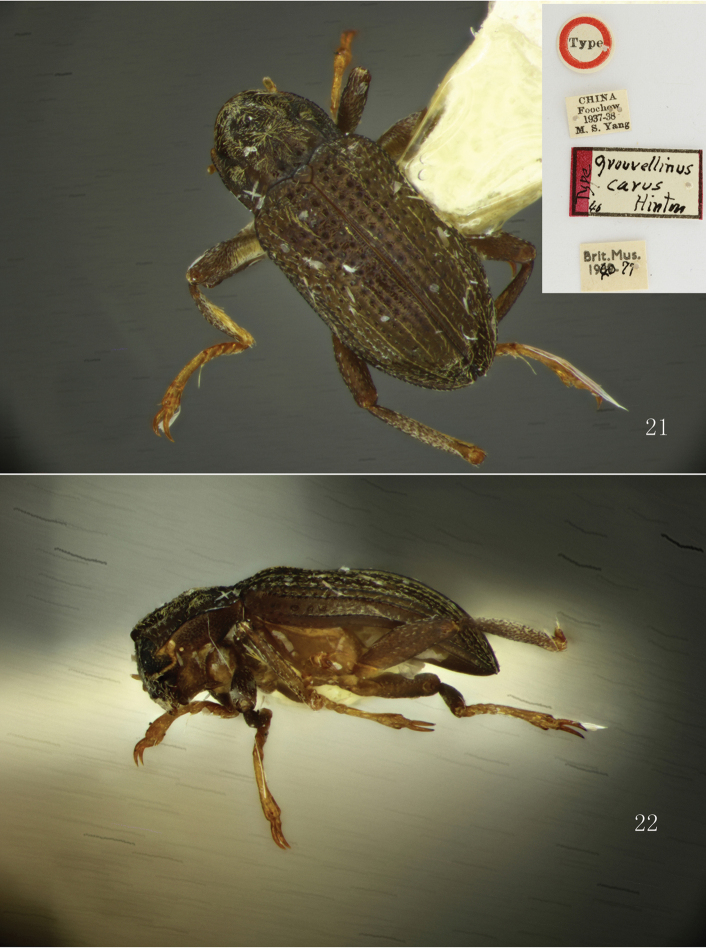
*Grouvellinus carus* Hinton, 1941, holotype. **21** habitus, dorsal view **22** lateral view. Photograph by Keita Matsumoto (London).

**Figures 23–28.**
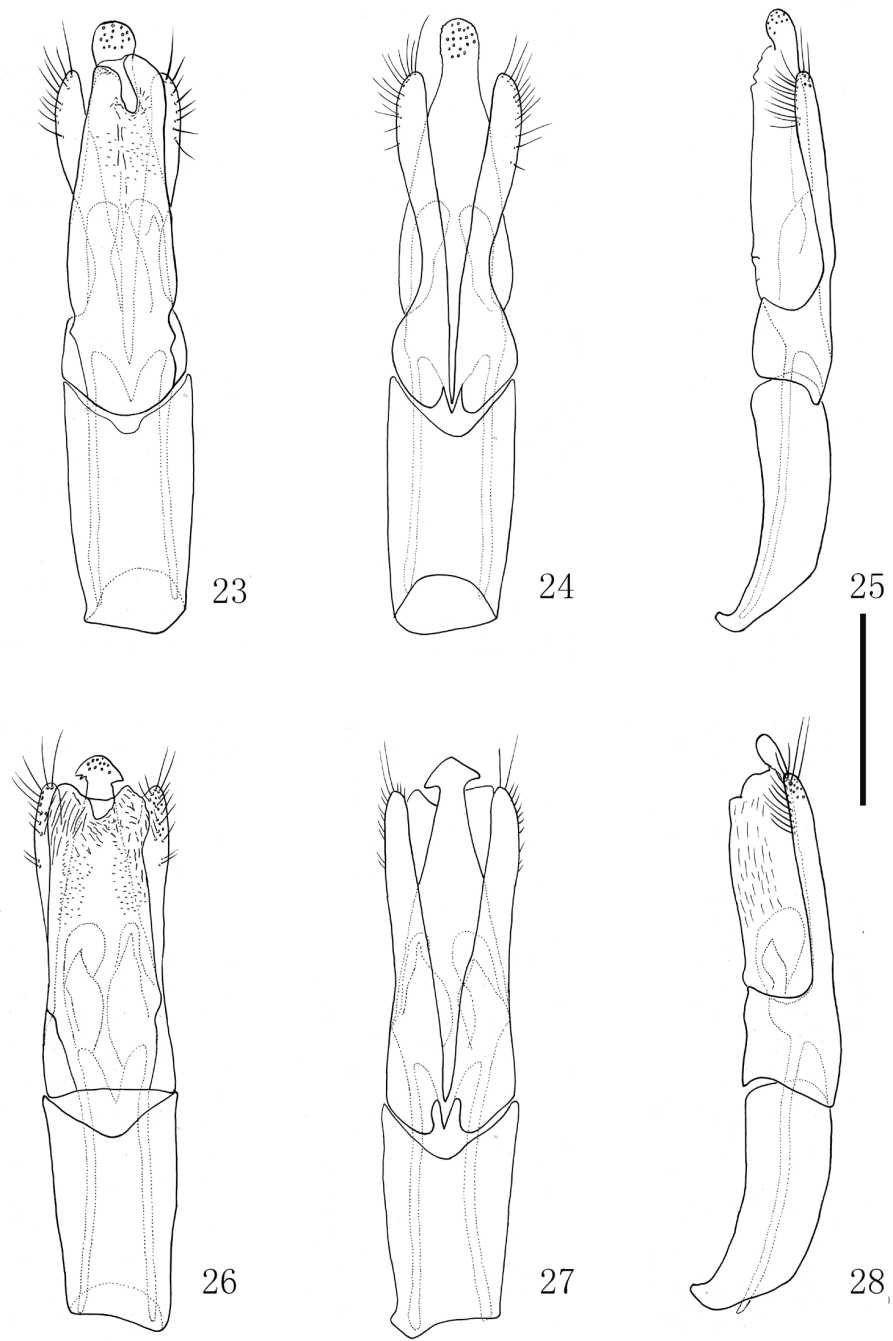
Male genitalia. **23–25** *Grouvellinus orbiculatus* sp. n., holotype **26–28** *Grouvellinus sagittatus* sp. n., holotype **23, 26** in ventral view **24, 27** in dorsal view; **25, 28** in lateral view. Scale bar: 0.2 mm.

## Concluding remarks

The focus of this study, is on the species of *Grouvellinus* which occurr in mainland China and Taiwan. Ten species including the two new species were examined. Some species occurring in neighboring countries such as *Grouvellinus brevior* (Nepal), *Grouvellinus nitidus* Nomura (Japan), and *Grouvellinus subopacus* Nomura (Japan) were also examined during the study. Habitus and male genitalia photos of *Grouvellinus sculptus* Bollow, 1940 (Myanmar) were provided by Dr. Johannes Bergsten (Stockholm).Unfortunately, we were unable to examined *Grouvellinus carus* Hinton and *Grouvellinus chinensis* Mařan. The original description of *Grouvellinus carus* was detailed and habitus photos were clear, so some differences between *Grouvellinus carus* and the two new species could be observed. The type specimen *of Grouvellinus chinensis* was not deposited in the National Museum, Prague, Czech Republic (NMPC) as mentioned in the original publication, so the new species could only be compared with the original description of *Grouvellinus chinensis*, but the original description was simple, so the main differences observed were the body size and carinae on elytral intervals. The topotype of *Grouvellinus chinensis* Maran should be collected improving the revison of *Grouvellinus*.

## Supplementary Material

XML Treatment for Grouvellinus orbiculatus

XML Treatment for Grouvellinus sagittatus

XML Treatment for Grouvellinus babai babai

XML Treatment for Grouvellinus chinensis

XML Treatment for Grouvellinus hydropetricus

XML Treatment for Grouvellinus montanus

XML Treatment for Grouvellinus nepalensis

XML Treatment for Grouvellinus pilosus

XML Treatment for Grouvellinus sinensis

XML Treatment for Grouvellinus hercules

XML Treatment for Grouvellinus tibetanus

XML Treatment for Grouvellinus carus
